# A Systematic Review of Psychobiotic Interventions in Children and Adolescents to Enhance Cognitive Functioning and Emotional Behavior

**DOI:** 10.3390/nu14030614

**Published:** 2022-01-30

**Authors:** Melissa Basso, Nicola Johnstone, Paul Knytl, Arjen Nauta, Andre Groeneveld, Kathrin Cohen Kadosh

**Affiliations:** 1 Department of Psychological Sciences, School of Psychology, Faculty of Health and Medical Sciences, University of Surrey, Guildford GU2 7XH, UK; m.basso@surrey.ac.uk (M.B.); p.knytl@surrey.ac.uk (P.K.); 2 FrieslandCampina, 3818 LE Amersfoort, The Netherlands; arjen.nauta@frieslandcampina.com (A.N.); andre.groeneveld@frieslandcampina.com (A.G.)

**Keywords:** psychobiotics, probiotics, prebiotics, children, adolescents, dietary interventions

## Abstract

This systematic review brings together human psychobiotic interventions in children and adolescents (aged 6–25 years) to evaluate the efficacy of pre- and probiotic supplements on stress, anxiety, and cognitive outcomes. Psychobiotic interventions in animal studies highlighted sensitivity to effects during development and maturation in multiple domains from emotion to cognitive processing. Several translational psychobiotic interventions in humans have been carried out to assess effects on emotion and cognition during childhood and into adulthood. The findings illustrate that there are limited consistent psychobiotic effects in developing human populations, and this is proposed to be due to heterogeneity in the trials conducted. Consequentially, it is recommended that three specific factors are considered in future psychobiotic trials: (1) Specificity of population studied (e.g., patients, developmental age), (2) specificity of intervention, and (3) homogeneity in outcome measures.

## 1. Introduction

In the last decade, research has repeatedly highlighted the key role that gut microbiota play in regulating the brain, cognition, and subsequent behavior in mature adults [1,2,3] and in childhood and adolescence [4,5,6] The human gut microbiota is composed of bacteria, archaea, yeasts, viruses and protozoa [7] and the term “gut microbiome” specifically refers to the “the genes and genomes of the microbiota, as well as the products of the microbiota and the host environment” [8]; see also [9] for a critical discussion. The gut microbiota and human host act as a holobiont, codependent for survival and homeostatic balance [10,11,12]. This is an essential state for maintaining gastrointestinal and metabolic functions and brain and behavior regulation [1,13]. For example, humans’ gut microbiota is shaped via diet choices [14], and the gut microorganisms reciprocally contribute to host physiology maturation and maintenance [15,16].

Bidirectional communications between the gut and brain (the so-called gut–brain axis, GBA) [17,18,19] allow gut microbiota to regulate gene expression in the brain by inhibiting micro-RNA (miRNA) and messenger RNA (mRNA) translation [20]. The increased or decreased expression levels of many miRNAs reflect various pathophysiologic processes of diseases via the synthesis and release of neurotransmitters implicated in psychopathology, such as gamma-aminobutyric acid (GABA) and the precursor pool for serotonin, [21], as well as brain-derived neurotropic factor expression [22]. Neuroactive metabolites produced by bacteria directly interact with gut autonomic synapses [23], and ultimately modulate brain neurochemistry and behavior. Bacteria also act via the production of short-chain fatty acids (SCFAs) and peptidoglycan (PGN). SCFAs enter circulation and have widespread effects on the brain directly (e.g., [24]) and the mucosal immune system [25].

Evidence in animal models found gut microbiota influences initial brain development, including synaptogenesis and myelination of brain areas [4], and brain responsiveness and function across the lifespan [26]. Bacterial colonization in human infants begins at birth [27], and over several years diversifies in richness and functional capacity [28]. Gut microbiota composition continues to adapt during development, characterized by a relative abundance of genes that support functional and structural brain development, maturing into adulthood with genes that reflect the host state (e.g., in disease, there is a greater predominance of genes related to inflammation, obesity, or adiposity) [29].

**Figure 1:**
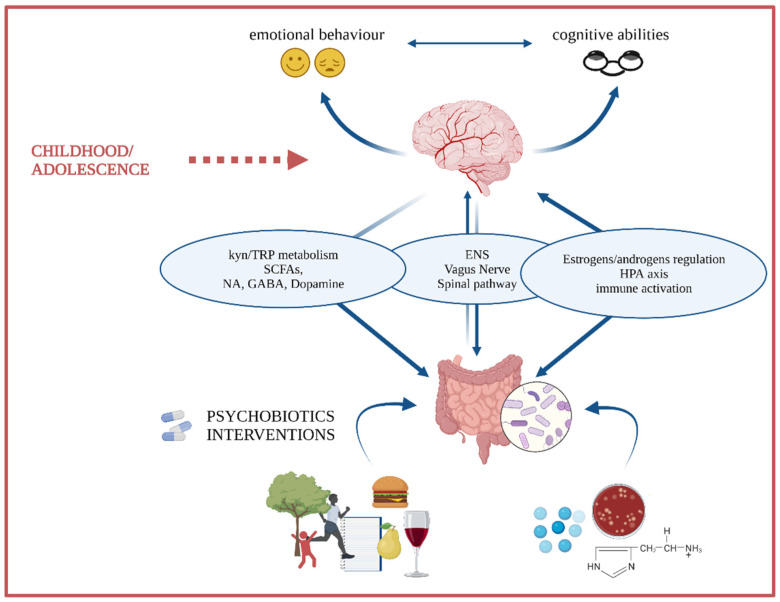
During childhood and adolescence, the individual is likely to undergo significant internal and external environmental changes and demands. At the same time, this is a time window when neuroplasticity is enhanced, allowing brain structures and circuitries to flexibly adapt or maladapt to the environment. In this context, gut microbiota might be a mediator between the environment and the CNS via multiple pathways that include: (i) the vagus nerve and spinal tract, whose action can be either direct or mediated by the ENS; (ii) the HPA axis; (iii) sex hormones (e.g., estrogens and androgens); (iv) microbes’ production of proinflammatory compounds, which can lead to systemic inflammation and microglia activation; and (v) microbes’ metabolites able to cross the BBB (e.g., SCFAs) and to alter the tryptophan/kynurenine pathways. Gut microbiota can be easily manipulated through diet; thus, it could be a promising therapy target in the redirection of neurodevelopmental trajectories.

To date, research has focused on characterizing microbe populations in health and disease to discover avenues for intervention [7,17,30,31,32]. Specifically, animal and human research has pointed towards the transitional period from mid-childhood to early adulthood (approximately 10–25 years) as a sensitive time window during which the microbiota gut–brain axis is fine-tuned [33]. This critical time window coincides with ongoing maturation and increased plasticity levels at the behavioral and brain levels [34,35,36], encompassing a period sensitive to developing mental health problems. Therein, the gut microbiome might be a key mediator between the environment and the developing brain via multiple pathways (Figure 1). As the gut microbiota is easily manipulated through diet and food supplements (e.g., prebiotics and probiotics), this represents a promising target for shaping the microbiota GBA, and thus for redirecting neurodevelopmental trajectories. Critically, this would be important for better cognitive functioning and well-being in development.

### Targeting the Microbiota Gut–Brain Axis to Improve Developmental Outcomes

Microbial ecology is modified therapeutically via the intake of so-called psychobiotics to help reduce stress responses and symptoms of anxiety and depression [31,37,38], as well as to increase cognitive functioning [14,39]. The term psychobiotics refers to live cultures of beneficial gut bacteria (probiotics) or substrates from fibers (prebiotics) that enhance the growth and/or activity of indigenous beneficial intestinal bacteria, which can improve brain function [40,41].

Probiotic strains, including members of the genera *Lactobacillus* and *Bifidobacterium*, are enriched in some dairy/fermented products, whereas prebiotics are nondigestible substances that feed the gut microbiome [42,43], such as oligosaccharides found in in cereals, fruits, and vegetables [40]. Both pro- and prebiotics are also commercially available as supplements. Both affect emotional, cognitive, systemic, and neural measures of anxiety in healthy and clinical populations [44,45]; see also [46,47,48] for systematic reviews of the evidence in adults and young people. Importantly, if administered during childhood and adolescence, probiotics and prebiotics might optimally act on cognition, anxiety, and maladaptive stress responses [49,50]. To date, there are limited summaries interrogating the evidence for efficacy of psychobiotics to improve adaptive functioning in both children and young people across cognitive and emotional domains. Moreover, no systematic overview is available that looks at the effects of factors such as timing and dosage on the various outcome measures.

## 2. Objectives

We systematically reviewed current evidence of psychobiotic interventions in children and adolescents to improve well-being and cognitive functioning to: (1) describe the efficacy of psychobiotics to influence and improve emotional well-being and cognitive functioning in childhood and adolescence, and (2) develop evidence-based recommendations for future research and intervention approaches.

## 3. Methods

### 3.1. Protocol

A systematic search was performed on trials in humans aged 6–25 years in which an active treatment (probiotics or prebiotics) and placebo were included. Primary outcomes were anxiety symptomatology and cognitive functions, and the secondary outcome was stress. The reporting of methods was consistent with the Preferred Reporting Items for Systematic Reviews and Meta-analyses (PRISMA) guidelines [51] and recommendations of the Cochrane Collaboration [52] (for the PRISMA checklist, see supplemental material). This protocol was registered on PROSPERO on 16 November 2020, accessible at https://www.crd.york.ac.uk/PROSPERO/display_record.php?RecordID=213265 (accessed 1 November 2021). All materials and data collected were reported.

### 3.2. Eligibility Criteria

Controlled trials assessing anxiety and/or stress, or cognition with at least one active treatment group and one comparator group in both subclinical and clinical populations were included. Inclusion criteria were: (1) mean age in the range of 6–25 years old, (2) healthy and clinical samples, (3) minimally measures obtained pre- and postintervention, (4) pro- or prebiotic administration in any form, (5) anxiety or cognition measured as primary or secondary outcomes with stress proxies also included when present, (6) employment of validated measurement instruments, (7) published and peer-reviewed data, and (8) any date of publication. Exclusion criteria were: (1) administration of a pro- and prebiotic combination (i.e., synbiotics) to avoid any confusion due to interaction effects (2) duplicate data/publications, and (3) unpublished data to ensure good research quality.

### 3.3. Search Strategy, Study Selection, and Data Extraction

Six databases were searched (PubMed, Embase, Cochrane, Scopus, Ovid, and Web of Science) between 18 and 27 October 2020 using the search terms reported in the supplemental material with no date of publication restrictions, in addition to hand-searching bibliographical articles associated with mental health. Searches for anxiety and/or stress and cognition were conducted in parallel, and outputs were imported into EppiReviewer4 V 4.11.5.3 [53], and duplicates were removed. Titles and abstracts were double screened before full-text articles were retrieved and screened. Spreadsheets were used to extract: (1) the first author’s surname and the year of publication; (2) samples size, mean age, gender, and population; (3) type and length of intervention, dose, frequency, and delivery method; (4) comparator; (5) assessment methods of outcomes; and (6) outcome results as reported by the included studies. Authors were contacted when data were missing. Data were qualitatively synthesized as too heterogeneous to perform any statistical summary.

### 3.4. Risk of Bias Assessment

The Revised Cochrane risk-of-bias tool for randomization trials (RoB-2) [54] was used to consider the following bias domains: (1) random sequence generation (selection bias), (2) allocation concealment (selection bias), (3) personnel and participant blinding (performance bias), (4) outcome assessment blinding (detection bias), (5) incomplete outcome data (attrition bias), (6) selective reporting (reporting bias), and (7) other sources of bias.

## 4. Results

### 4.1. Stress/Anxiety

#### 4.1.1. Study Records

**Figure 2:**
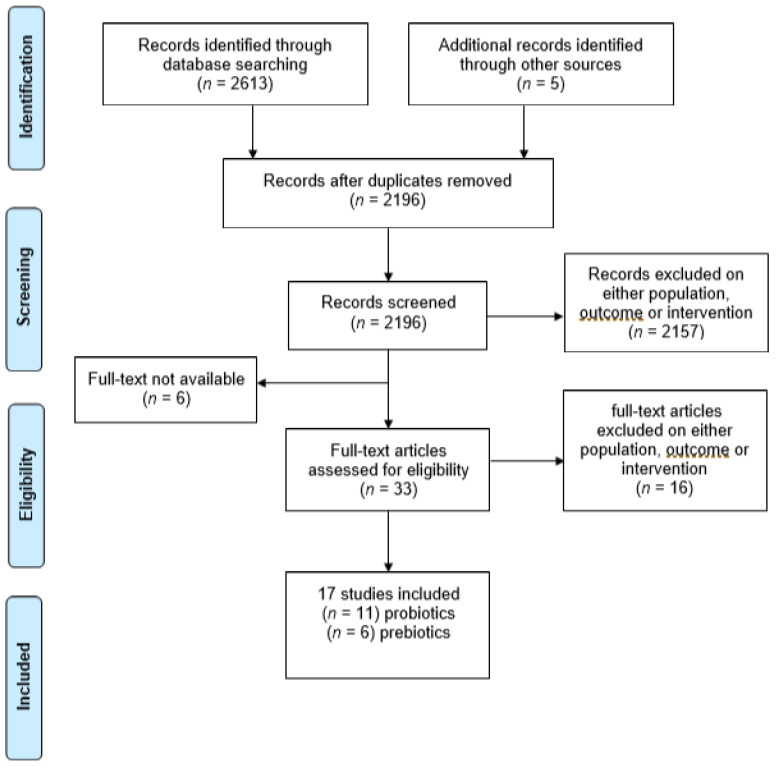
PRISMA flowchart of search results of each step of the review for anxiety/stress outcomes.

The search identified 2618 relevant studies. After removal of 422 duplicates, 2196 abstracts and titles were double screened. Then, 2157 were excluded for ineligible samples, outcomes, or intervention, and 6 for text unavailability after request. A total of 33 full-text studies remained for eligibility assessment, of which 16 were excluded on either sample, outcome, or intervention. The final output was 17 studies—11 using probiotic interventions, and 6 using prebiotic interventions (Figure 2).

#### 4.1.2. Included Studies Characteristics

A complete summary of study [55,56,57,58,59,60,61,62,63,64,65,66,67,68,69,70,71] characteristics is depicted in Table 1. Among the 11 studies that employed probiotics, a variety of species and strains were used: *Saccharomyces boulardii*, *Lactobacillus casei Shirota*, *Lactobacillus plantarum DR7* or *PS128,* and *Lactobacillus rhamnosus* were administered singularly; or a combination of *Lactobacilli*, *Bifidobacteria* and/or *Streptococcus*. These were delivered either as capsules, powder, or sachets with a daily dose up to 1 × 10^11^ colony-forming units (CFUs) and a length of intervention ranging from 14 to 56 days. Concerning probiotic, six studies were identified, and galacto-oligosaccharides (GOS) and fructooligosaccharides (FOS) were the most used, followed by omega-3-polyunsaturated fatty acids (PUFAs) and fermented ginseng (FG). Omega-3-polyunsaturated fatty acids (PUFAs) and fermented ginseng (FG) were included due to their prebiotic effect on the gut microbiome [72,73]. Total daily dosage ranged between 18 and 5500 mg, for a minimum of 8 to a maximum of 84 days. Fourteen studies included a healthy sample of participants (e.g., students), either under stress conditions [57,58,59,60,61,63,64,67,71] or normal daily circumstances [55,56,62,68,69]. The included clinical populations were also highly heterogenous; that is, individuals were affected by anorexia nervosa [66], autism spectrum disorder (ASD) [65], or learning disabilities [70] with no anxiety diagnosis. Finally, a range of assessment tools were used to measure both anxiety (State-Trait Anxiety Inventory, Beck Anxiety Inventory, Child Behavior Checklist) and stress (cortisol, metanephrine, self-reported stress, heart rate), thus further contributing to the heterogeneity of the different constructs being studied.

#### 4.1.3. Quality of the Included Studies

Among the 17 included records, only 1 showed a low risk of bias [64], whereas 6 studies showed a high risk of bias [60,62,63,66,69,71] (see Figure S1). Reasons for moderate concerns in study designs were mostly related to the absence of pre-specified protocols to compare with the published reports and the absence of detailed and clear information about the randomization process. Kelly and colleagues [63] were assessed at high risk of bias due to design, and neither described as blinded nor double blinded, in addition to absent information regarding intervention adherence and missing data. The high risk of bias for Kato-Kataoka and colleagues and colleagues [61] was due to the randomization process and significant differences in baseline State-Trait Anxiety Inventory (STAI) scores. Similar reasons applied to Kitaoka and colleagues and colleagues [63] and Manos and colleagues and colleagues [66], although baseline differences were not significant. Other reasons for a high risk of bias were lacking details about protocol adherence/deviations and inappropriate analyses to account for drops-out as well as, in the case of Marcos and colleagues and colleagues [70], concerns about the design, which was described as neither blinded nor double-blinded.

#### 4.1.4. Intervention Effects

Four studies found significant results in anxiety or stress outcomes using a probiotic intervention. A 28-day multi-probiotic intervention significantly decreased worrying measured by the Penn State Worry Questionnaire (PSWQ) [62] whereas 84 days of *L. plantarum* administration led to improvements in the Depression Anxiety Stress Scale (DASS-42), both in anxiety and stress scores [56]. On the other hand, following *L. casei Shirota* intervention, no effects on anxiety were reported [60,61] but were evident for stress assessed via a visual analogue scale (VAS) and cortisol levels [58]. Similarly, self-reported stress measures were shown to ameliorate after 42 days of supplementation with *L. helveticus* [57].

Regarding prebiotics, only two studies reported outcome improvements, a reduction in the Beck Anxiety inventory (BAI) scores following 84 days of omega-3 supplementation [64]; and second, decreased attentional bias towards negative emotion stimuli, accompanied by decreased cortisol levels after 21 days of GOS intervention [68]. It is worth mentioning that Kitaoka and colleagues [62] reported a within-group decrease in total anxiety scores following 8 days of fermented ginseng supplementation, although no information about group comparison was explicitly reported by the authors.

### 4.2. Cognition

#### 4.2.1. Study Records

**Figure 3:**
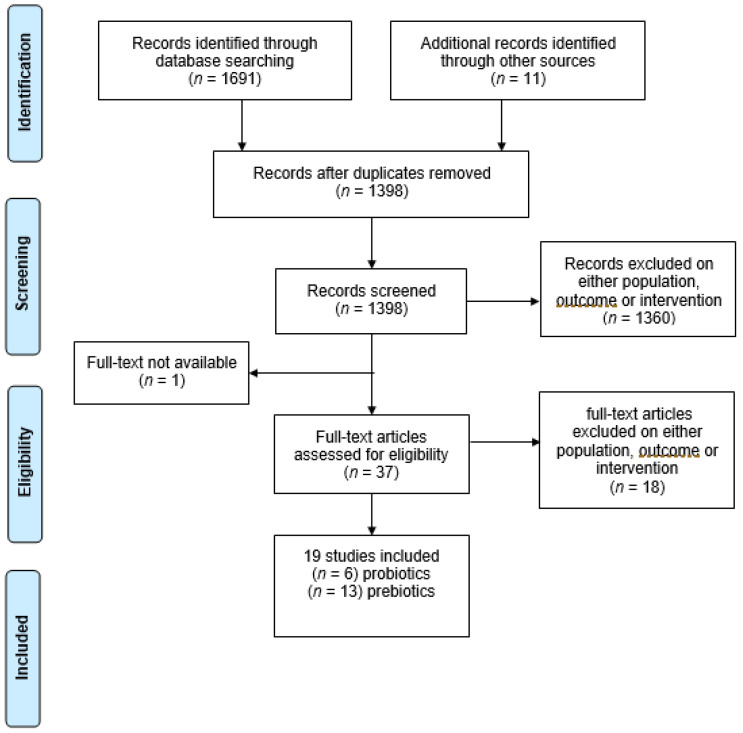
PRISMA flowchart of search results of each step of the review for cognitive outcomes.

The search identified 1702 studies. After 304 duplicates were removed, 1398 abstracts and titles were double screened. 1360 were excluded for ineligible samples, outcomes, or intervention, and 1 for text unavailability. A total of 37 full-text studies remained for eligibility assessment, of which 18 were excluded on either sample, outcome, or intervention. The final output for the systematic review was 19 studies, 6 using probiotic interventions and 13 using prebiotic interventions (Figure 3).

#### 4.2.2. Included Studies’ Characteristics

A summary of study [55,56,63,65,67,68,74,75,76,77,78,79,80,81,82,83,84,85,86] characteristics is depicted in Table 2. Among the six studies that employed probiotics, a variety of probiotic species and strains were used: *Lactobacilli casei Shirota*, *Lactobacillus plantarum DR7*, *Lactobacillus plantarum PS128*, and *Lactobacillus rhamnosus*, as well as a combination of *Bifidobacterium bifidum W23*, *Bifidobacterium lactis W51*, *Bifidobacterium lactis W52*, *Lactobacillus acidophilus W37*, *Lactobacillus brevis W63*, *Lactobacillus casei W56*, *Lactobacillus salivarius W24*, *Lactococcus lactis W19*, and *Lactococcus lactis W58* (i.e., the Ecologic Barrier formula). Delivered using the same methods of the anxiety studies, probiotic dosages ranged between 1 × 10^9^ CFU and 3 × 10^10^ CFU administered once or twice a day, whereas the intervention length was between 28 and 84 days. In contrast, prebiotic supplementation used mostly PUFAs, such as docosahexaenoic acid (DHA) and eicosapentaenoic acid (EPA), with some using GOS or FOS. Total daily dosage ranged between 400 mg [78] and 5500 mg [68] for a minimum of 21 and a maximum of 121 days. Most probiotic studies were conducted on healthy participants, whereas prebiotics studies involved clinical samples, including children with attention-deficit hyperactivity disorder (ADHD), learning disabilities, and mood disorders. Cognitive functions measured included literacy, assessed by British Ability Scale-III; inhibition (e.g., go/no-go task), executive functions (e.g., Behavior Rating Inventory of Executive Functions), memory (e.g., digit span—forward/backward), cognitive reactivity (Leiden Index of Depression Sensitivity—revised) and attention (e.g., attentional dot-probe task).

#### 4.2.3. Quality of the Included Studies

Among the 19 included records, 5 showed a low risk of bias [76,79,80,81,83] and 2 a high risk of bias [63,85], while the remainder raised some concerns in bias (Figure S2). Reasons for moderate concerns in bias and high risk of bias in Kelly and colleagues and colleagues [63] were the same as reported for the anxiety studies. Additionally, the risk of bias for Vesco and colleagues and colleagues [85] was assessed as high due to the absence of justifications for the multiple imputation model in correcting for missing data in addition to an absence of reported baseline measures and a high probability that numerical results were selectively reported.

#### 4.2.4. Intervention Effects

Only two out of six interventional studies using probiotics found significant effects. Specifically, following 56 days of *L. casei Shirota* administration, sustained attention was shown to improve in young football players, as indexed by a decrease in reaction times in the digit vigilance test [55]. This was in contrast to Liu and colleagues [65], who did not find any improvement in parental-reported attention scores in children affected by ASD. While both Chong and colleagues [56] and Kelly and colleagues [63] did not find any significant treatment effects, Papalini and colleagues [67] found improved working memory in the digit span-backward test following 28 days of a multispecies probiotic administration, although only under acute psychophysical stress. Notably, the same intervention ameliorated cognitive reactivity to sad mood in depressed participants [84].

Regarding prebiotics, 8 out of 13 studies reported significant results. Attention was shown to improve in Bos and colleagues [74] in ADHD participants following DHA and EPA administration, and in healthy participants under GOS supplementation [68]. In contrast, no attentional improvements were found in children with ADHD who were administered DHA only [86]. Heterogenous results were also found for inhibitory control measures, with significant improvement following 91 days of DHA and EPA administration in children with moderate ADHD [76], whereas no effects were found in ADHD children [74] nor in college students [77]. In contrast, in Karr and colleagues [77] the placebo group presented increased executive control. Studies investigating general cognitive functions [78,82] showed positive effects of DHA and EPA combined and DHA alone in healthy participants, improved executive functions in children with mood disorders [85], and increased working memory scores in ADHD participants [81]. Concerning literacy, neither PUFAs nor GOS had consistent effects in ADHD participants [79,80], whereas Richardson and colleagues [83] reported improved reading scores in poor readers but otherwise healthy children.

### 4.3. Overall Results

**Figure 4:**
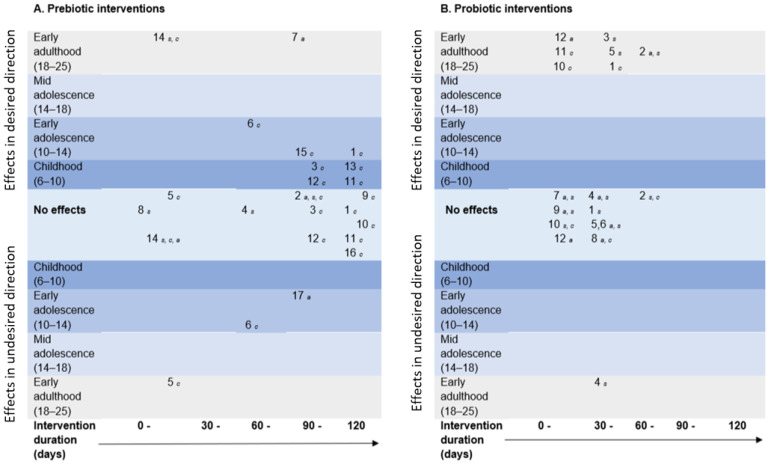
Mapping of systematic review outcomes by mean age of participant sample (x-axis, numbers in brackets refers to the age range) and intervention duration (y-axis) for each included study in which outcomes were categorized as effects in the desired direction (e.g., decreased anxiety, stress, or improved cognition vs. placebo), effects in the undesirable direction (e.g., increased anxiety, stress, or diminished cognitive effects vs. placebo), or no effects for pre- (**A**) and probiotic (**B**) interventions separately. Numbers in each panel refer to each specific study (indexed below), and letter subscripts refer to the categorical outcome (specified below). The key for each panel is as follows. (**A**). Studies using prebiotic interventions: ^1^ [74],^2^ [70], ^3^ [76],^4^ [58], ^5^ [77], ^6^ [78], ^7^ [64], ^8^ [62], ^9^ [79], ^10^ [80], ^11^ [81], ^12^ [82], ^13^ [83], ^14^ [68], ^15^ [85], ^16^ [86], ^17^ [66]. *a =* anxiety outcome, *s* = stress outcome, *c* = cognition outcome. (**B**). Studies using probiotic interventions: ^1^ [55], ^2^ [56], ^3^ [57], ^4^ [59], ^5^ [60], ^6^ [61], ^7^ [63], ^8^ [65], ^9^ [71], ^10^ [67], ^11^ [84], ^12^ [69]. *a =* anxiety outcome, *s* = stress outcome, *c* = cognition outcome.

Figure 4 maps efficacy in the available qualifying evidence. In most cases, prebiotic interventions were longer, delivered to subjects younger than 14 years of age, and targeted to improve cognition. In contrast, probiotics were mostly administered to young adults, with a shorter duration and a focus on improving anxiety and stress. Illustratively, there was limited evidence for psychobiotics’ efficacy, and most positive responses to intervention were found for cognitive outcomes.

## 5. Discussion

This systematic review aimed to describe the current evidence for the effectiveness of prebiotic- and probiotic-based interventions in the management of stress and anxiety, and in improving cognition in human children and adolescents, in addition to making recommendations for future research and intervention approaches. Based on the current findings, evidence available to support the use of psychobiotics for anxiety, stress, and cognition in therapeutic interventions in children and adolescents is minimal.

### 5.1. Anxiety and Stress Findings

From 17 eligible studies (11 probiotic), there is currently limited evidence for the efficacy of using probiotic and prebiotic interventions in alleviating anxiety and stress responses in children and adolescents. Several factors influenced this outcome; for example, heterogeneity in treatments, dosage, and duration rendered direct intervention comparisons difficult. Similarly, conceptualization of outcome measures varied across studies, complicating evaluation of anxiety and stress indices. This was further acerbated where full-texts or effect sizes (and raw data) were unavailable and not provided after request. Notably, whereas most studies used probiotic supplements for the intervention, two prebiotic studies (including PUFAs) (e.g., [64,68]) found effects on anxiety and stress measures, warranting further investigation. Specifically, as the reported effects might be underpinned by microbiota growth action and/or anti-inflammatory and immunomodulatory mechanisms, future human studies should use stool samples and biological proxies to better delineate PUFAs’ mechanisms of action.

### 5.2. Cognitive Findings

A total of 19 eligible studies (13 prebiotic) evaluated cognitive outcomes. Therapeutic psychobiotic use for cognitive improvements in development is encouraging, with 50% of studies reporting success. However, considerable variations in study design, intervention protocols, and participants sampled prevented a meta-analysis of the obtained effects. This was compounded by the range of cognitive functions investigated, including diverse measures in broad domains conceptualizing attention, executive functions, or working memory.

These results agreed with a recent systematic review and meta-analysis of pre- and probiotics and fermented foods on cognitive outcomes in human adults. Marx and colleagues [87] found more than half of eligible studies reported improved cognitive outcomes as a function of active intervention, yet this was not represented in the meta-analysis showing no detectable effects. Similarly, another systematic review of probiotic effects on cognition in all ages identified probiotic driven improvements in cognition in the majority of studies, yet highlighted problems in the quality of the studies [48]. Both studies commented on study quality, with problems in heterogeneity in clinical presentations, in addition to small samples and short-term interventions. These methodological issues were also present in younger age groups.

An observation on domain-specificity of psychobiotic intervention effects was drawn during this review; specifically, whether improvements in one domain (e.g., cognitive functions) would generalize to improvement in other measured domains (e.g., anxiety and/or stress). Several included studies facilitated this observation with the inclusion of both cognitive and anxiety/stress measures [55,56,63,65,67,68,70]. This subsample illustrated one occurrence of both cognitive and emotional improvements after 4 weeks of prebiotic supplement intake [68]. The remaining studies used probiotic interventions with emotional and cognitive measures, and found improvements in cognitive functions [55] or anxiety/stress indices [56] independently, or no improvements at all [63,65]. This observation suggested that prebiotic interventions might have a broader effect on overall functioning (domain general), whereas probiotic interventions may be more targeted in their effects. Mechanistic actions of pre- and probiotics largely overlap, and differentiating pathways to effects on the GBA in humans is further compounded by known external influences of the environment and dietary choices. More research is now needed to elucidate the utility of specific pre- and probiotic effects.

### 5.3. Towards a More Standardized Research Approach in the Field of Psychobiotic Interventions

**Figure 5:**
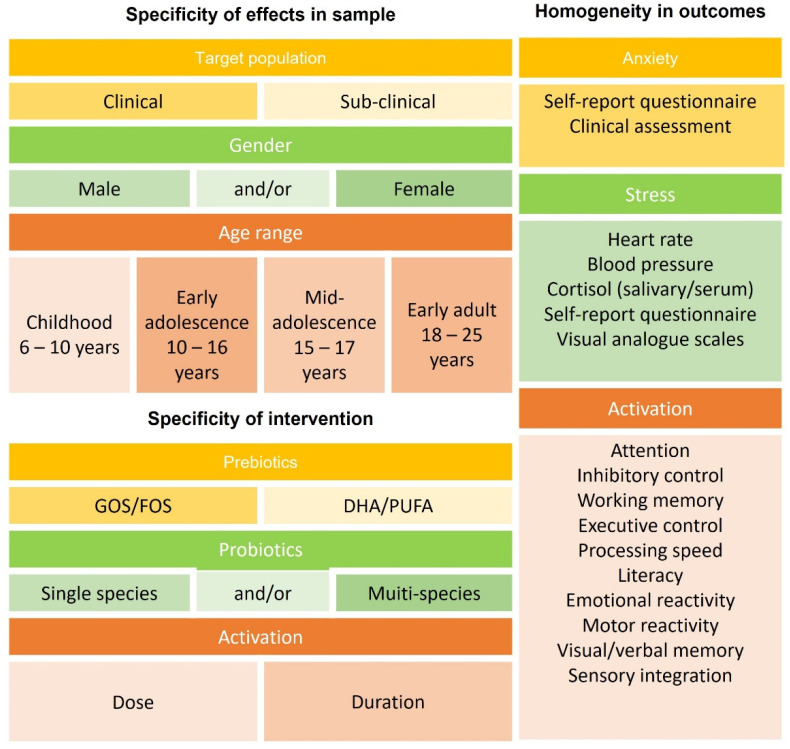
Key recommendations for future psychobiotic interventions, based on observations from this study. Recommendations can be considered in three broad aspects: specificity of effects in the sample selected, specificity of effects from the intervention, and homogeneity in outcomes. The broad wash of outcomes in psychobiotic intervention trials with human participants was due to heterogeneity in the trials conducted and the key variables highlighted here. For population level effects to be established, it is recommended that future trials situate research in consideration of sample, intervention, and outcome.

A final objective of this study was the development of specific recommendations for future research. Based on the evidence reviewed, stringent standards are required to systematically advance this field. Key elements that must be considered are outlined in Figure 5.

Apart from extending the research approach to both preclinical and clinical populations, the most important improvement is the adoption of a standardized intervention protocol in terms of dosage and intervention length for both probiotics and prebiotics deployed. For example, probiotic intervention dosages were similar across cognition and anxiety/stress studies (cognition: 1 × 10^9^–3 × 10^10^ CFU; anxiety/stress: 1 × 10^9^–3 × 10^10^ CFU), whereas prebiotic interventions varied in dosage, with cognition studies using a lower dosage (400–1467 mg, except for Schmidt and colleagues [68], which employed 5500 mg) in comparison to anxiety/stress studies (1845–5500 mg). This could be related to the prevalence of PUFA administration within the cognitive domain, an active ingredient for which a lower dosage might be sufficient for efficacy in comparison to GOS.

Dosage requirements could differ by age; for example, children seem to not benefit from higher dosages (and intervention lengths) of DHA and EPA [79,80,86] in comparison to young adults [77]. Based on these findings, the standardization of intervention protocols should be tailored to age/developmental differences to allow comparison across studies employing the same active ingredient (e.g., PUFAs), and across compounds (e.g., PUFAs and GOS).

We note that not all supplements labelled prebiotics in the current systematic review are currently recognized as prebiotics. Specifically, we included several studies that used DHAs and PUFAs to improve either cognitive or anxiety indices. The rationale for this approach was that research has shown that DHAs and PUFAs mechanistically have a prebiotic effect on the gut microbiome [72], and therefore operate as functional psychobiotics.

The target populations for psychobiotic interventions also needs to be considered; we have highlighted the potential for differential developmental effects, and this encapsulates neurodiversity as well. For example, children with neurodevelopmental disorders such as autism and ADHD frequently present with comorbid gastrointestinal problems. Psychobiotics (especially PUFAs) seem promising for improving outcomes in those with ADHD. High-quality studies in developing populations with clinical neurodevelopmental disorders might be particularly beneficial in improving gastrointestinal and cognitive and/or emotional outcomes.

Similarly, comparable intervention lengths would provide time for additional follow-up testing points to assess the longevity of the effects. Critically, attention should be paid to the fact that effects on anxiety seem to appear with longer interventions [55,63] than stress [57,60,68], suggesting that anxiety improvements might be secondary, or dependent on changes to the physiological equilibrium. Standardized protocols would allow direct comparison of the effectiveness of different prebiotics and probiotic strains, and provide the foundation for further customize therapeutic intervention protocols for different age groups and conditions. Additionally, it would be beneficial to include measures of gut microbiome composition at both baseline and postintervention time points (e.g., [44]) to assess whether the observed effects at the behavioral level can be linked to changes in the gut microbiome.

Moreover, given the reciprocal relationship of the gut microbiome and the human host, future studies should include additional measures of factors that have all been shown to influence both the gut microbiome and behavior, such as diet, sleep, and exercise. Comprehensive stakeholder engagement, such as focus group work with children, adolescents, and their parents, as reported in a recent study [46], may lead to the discovery of additional important factors that influence the success of these interventions. In addition, this important feedback from participants with lived experience of problems with cognitive functioning and/or emotional behavior will further help tailor intervention protocols to ensure high levels of treatment uptake and compliance.

Last, to aid with standardization and greater comparability of intervention effects across studies, it will be essential to work towards a greater agreement on behavioral measures and instruments for both cognitive and mental health indices. This is in line with current developments in the field of mental health research. For example, in June 2020, the National Institutes of Mental Health, USA, and the Wellcome Trust, UK (which are two of the largest funders for mental health research) announced plans for a more standardized mental health research approach [88]; see also [89] for a critical discussion.

## 6. Conclusions

To date, interventions on psychobiotic outcomes have shown some beneficial effects on anxiety, stress, and cognition via both prebiotic and probiotic intake; however, these were not consistent. This review illustrated that the inconsistency was due to heterogeneity in the trials conducted, and recommends consideration of three key factors in future psychobiotic trials: specificity of sample, specificity of intervention, and homogeneity in outcomes acquired. Further, it is recommended that psychobiotic interventions obtain metrics of gut microbiota composition and tangible factors relating to food intake and physical activity to help elucidate steering factors of psychobiotic effects. This is critical, as the term psychobiotics implies a proven effect of the pro- and prebiotic supplements on the gut microbiome and the brain; however, more research is required to draw out consistency in the effects.

## Figures and Tables

**Table 1:** Characteristics of the anxiety/stress studies included in the systematic review.

Study	Intervention Type	Delivery Method	Active Compound	Dose	Frequency (Dose/Day)	Duration (Days)	Active/ Control	Mean Age	Sex (M/F)	Anxiety Measure	Effect WITHIN-AG	Effect BETWEEN-G	Stress Measure	Effect WITHIN-AG	Effect BETWEEN-G	Participants	Risk of Bias
Adikari et al., (2020) [55]	probiotic	liquid	Lactobacillus Casei Shirota	3 × 10^10^ CFU	1	56	10/9	19	19/0	-	-	-	EDR	-	ns	football players	
													HR	-	ns		
Capitao et al., (2020) [70]	prebiotic	sachet	B-GOS	-	1	84	17/18	8.84	24/11	STAIC	ns	ns	salivary cortisol	-	ns	children with below-average literacy skills	
Chong et al., (2019) [56]	probiotics	sachet	Lactobacillus Plantarum DR7	1 × 10^9^ CFU	1	84	27/32	24.8	-	DASS-42 anxiety	<	↓	PSS-10	<	ns	healthy young adults	
													DASS-42 stress	<	↓		
Culpepper et al. (2016) [57]	probiotic	capsule	Lactobacillus helveticus R0052	3 × 10^9^ CFU	1	42	145	19.9	209/372	-	-	-	self-reported stress	-	↓ for B. Bifidum only, only in sleep deprived students	students e.s.	
			Bifidobacterium longum ssp. infantis R0033				147										
			Bifidobacterium bifidum R0071				142										
			placebo				147										
Hughes et al. (2011) [58]	prebiotic	sachet	GOS	0, 2500, 5000 mg	1	56	279/140	19.9	207/212	-	-	-	self-reported stress	-	ns	students e.s.	
Karbownik et al. (2020) [59]	probiotic	capsule	Saccharomyces boulardii	5 × 10^9^ CFU	1	30	31/29	22.6	37/55	STAI state	<	ns	salivary cortisol	+	ns	students e.s.	
													salivary metanephrine	ns	ns		
													pulse rate	+	↑		
Kato-Kataoka et al. (2016) [60]	probiotic	liquid	Lactobacillus casei Shirota	100 × 10^9^ CFU	1	56	23/24	22.8	25/22	STAI state	-	ns	visual analogue stress scale	-	↓	students e.s.	
													salivary cortisol	-	↓		
													salivary alpha-amylase	-	ns		
Kato-Kataoka et al. (2016) [61]	probiotic	liquid	Lactobacillus casei Shirota	100 × 10^9^ CFU	1	56	24/23	22.9	26/21	STAI state	-	ns	salivary cortisol	-	ns	students e.s.	
													salivary immunoglobulin A	-	ns		
Kitaoka et al. (2009) [62]	prebiotic	capsule	Fermented Ginseng	205 mg	9	8	8/8	20.7	16/0	STAI total	<	-	salivary cortisol	ns	-	healthy subjects	
										POMS	ns	-	salivary immunoglobulin A	ns	-		
Kelly et al. (2017) [63]	probiotic	capsule	Lactobacillus rhamnosus	1 × 10^9^ CFU	1	28	15/14	24.6	29/0	BAI	ns	ns	PSS-10	ns	ns	healthy subjects, SECPT	
										STAI trait	ns	ns	cortisol SECPT	-	ns		
										STAI state	<	ns	self-reported stress SECPT	ns	ns		
Kiecolt-Glaser et al. (2011) [64]	prebiotic	capsule	omega-3 PUFAs	2500 g	1	84	34/34	23.7	38/30	BAI	-	↓	-	-	-	students e.s.	
Liu et al. (2019) [65]	probiotic	capsule	Lactobacillus plantarum PS128	30 × 10^9^ CFU	ns	30	36/35	10.0	71/0	CBCL	-	ns	-	-	-	ASD children	
Manos et al. (2018) [66]	prebiotic	capsule	omega-3-PUFAs	782 mg	4	84	10/8	14.7	0/18	BAIT	<	↑	-	-	-	anorexic girls	
Marcos et al. (2004) [70]	probiotic	liquid	Lactobacillus delbrueckii bulgaricus	1 × 10^9^ CFU	2	21	73/63	18–23	40/96	STAI state	+	ns	serum cortisol	-	ns	students e.s.	
			Streptococcus salivarius thermophilus	10 × 10^9^ CFU						STAI trait		ns					
			Lactobacillus casei DN114001	10 × 10^9^ CFU													
Papalini et al. (2019) [67]	probiotic	powder	Bifidobacterium bifidum W23, Bifidobacterium lactis W51, Bifidobacterium lactis W52, Lactobacillus acidophilus W37, Lactobacillus brevis W63, Lactobacillus casei W56, Lactobacillus salivarius W24, Lactococcus lactis W19, Lactococcus lactis W58	2.5 × 10^9^ CFU	2	28	29/29	21.5	0/58	-	-	-	VAS	+	ns	healthy subjects, SECPT	
													cortisol	+	ns		
													alpha-amylase	+	ns		
													HR	+	ns		
													BP	+	ns		
Schmidt et al. (2015) [68]	prebiotic	powder	FOS	5500 mg	1	21	15	23.7	22/23	STAI state	-	ns	PSS-10	-	ns	healthy subjects	
			B-GOS				15						salivary cortisol	-	↓ GOS only		
			placebo				15										
Tran et al. (2019) [69]	probiotic	-	18 species	50 × 10^9^ CFU (condition A)	1	28	14	20.6	20/66	BAI	-	ns BAI total	-	-	-	healthy students	
			10 species	50 × 10^9^ CFU (condition B)			13					-					
			18 species	15 × 10^9^ CFU (condition D)			15			PSWQ		↓50 × 10^9^ CFU only					
			10 species	10 × 10^9^ CFU (condition E)			15										
			placebo				11										
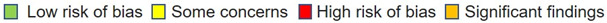																	

↓: Improvement vs. placebo; ↑: diminishment vs. placebo; <: decrease vs. baseline; +: increase vs. baseline; ns: no significant effect; -: not reported or not applicable; dose refers to amount of active compound. FOS: fructooligosaccharides; B-GOS: Bimuno^®^-galactooligosaccharides; PUFAs: polyunsaturated fatty acids; EDR: electrodermal activity; HR: heart rate; DASS-42: Depression Anxiety Stress Scale 42; BP: blood pressure; BAI: Beck Anxiety Inventory; CBCL: Child Behavior Checklist (Anxiety); CFU: colony-forming unit; PASAT: Paced Auditory Serial Addition Test; PSS: Perceived Stress Scale-10; PSWQ: Penn State Worry Questionnaire; STAI: State-Trait Anxiety Inventory; STAIC: State-Trait Anxiety Inventory for Children; e.s.: under examination stress, VAS: visual analogue scale; PSS-10: perceived stress scale; SECPT: socially evaluated cold pressor test; POMS: profile of mood states; ASD: autism spectrum disorders.

**Table 2:** Characteristics of the cognition studies included in the systematic review.

Study	Intervention Type	Delivery Method	Active Compound	Dose	Frequency (Dose/Day)	Duration (Days)	Active/ Control	Mean Age	Sex (M/F)	Cognitive Tool	Cognitive Function	Effect WITHIN-G	Effect BETWEEN-G	Participants	Risk of Bias
Adikari et al. (2020) [55]	probiotic	liquid (orange juice)	Lactobacillus Casei Shirota	3 × 10^10^ CFU	1	56	10/9	19	19/0	DVT-RT	sustained attention/vigilance/visual-motor tracking	+	↓	right-handed football players	
Bos et al. (2015) [74]	prebiotic	fortified margarine	DHA & EPA	650 mg each	1	112	19 ADHD PRO	10.6	76/0	CBCL-AP	attention	+	↓(ADHDvsRG) ↓(PROvsPBO)	ADHD children either medication naïve or using metylphenidate	
							19 ADHD PBO			GO/NO-GO TASK	inhibitory control	ns	ns		
							20 RG PRO								
							18 RG PBO								
Capitao et al. (2020) [75]	prebiotic	sachet	B-GOS	-	1	84	17/18	8.84	24/11	BAS-III	literacy	+	ns	children with below-average literacy skills	
										CogTrack	memory retrieval speed	+	ns		
Cornu et al. (2018) [76]	prebiotic	capsules	DHA & EPA	6–8 yo: 84 mg & 336 mg	1	91	71/77	9.9	127/35	Aloulette test	lexical age	-	ns	ADHD children	
				9–11 yo: 126 mg & 504 mg						KiTAP (6–10 yo)/TAP (11–15 yo)	distractibility (6–11 yo only)	-	ns		
				12–15 yo: 168 mg & 672 mg							flexibility	-	ns		
											inhibitory control (go/no-go RTs)	-	↓ RTs		
Chong et al. (2019) [56]	probiotic	sachet	Lactobacillus plantarum DR7	1 × 10^9^ CFU	1	84	27/32	24.8	-	CBB	psychomotor control	-	ns	stressed healthy subjects	
											basic attention	-	ns		
											visual learning & memory	-	ns		
											working memory	-	ns		
											executive function	-	ns		
											social emotional cognition	-	ns		
											associate learning	-	ns		
											verbal learning and memory	-	ns		
Karr et al. (2012) [77]	prebiotic	capsules	DHA & EPA	240 mg & 360 mg	2	28	20 /21	20.1	12/29	RAVLT	verbal learning and memory	ns	ns	college students	
										SCWT	inhibitory control	ns	ns		
										TMT	executive control	ns	↑		
Kelly et al. (2017) [63]	probiotic	capsules	Lactobacillus rhamnosus	1 × 10^9^ CFU	1	28	15/14	24.6	29/0	CANTAB	associate learning	+	ns	healthy subjects	
											attention	+	ns		
											visual speed processing	+	ns		
											emotional attentional bias	ns	ns		
Kennedy et al. (2009) [78]	prebiotic	capsules	DHA	200 mg	2 (400 mg)	56	28	10.8	44/42	CDR	general cognitive functions	+	↓word recognition speed in 400 mg pre&post breakfast ↑ in 1000 mg pre-breakfast	healthy children	
			DHA	200 mg	5 (1000 mg)		30			internet battery		-	-		
			PBO				30								
Liu et al. (2019) [65]	probiotic	capsules	Lactobacillus plantarum PS128	3 × 10^10^ CFU	-	30	36/35	10.01	71/0	CBCL-AP	attention	-	ns	ASD children	
Milte et al. (2012) [79]	prebiotic	capsules	DHA & EPA	108 mg & 1109 mg	1	121	24	8.9	70/17 *	WIAT-III/WSCI-III	literacy	-	ns	ADHD	
			DHA & EPA	1032 mg & 264 mg	1		19			TEAC	attention and inhibition	-	ns		
			safflower oil (control)	1467 mg	1		24								
Milte et al. (2015) [80]	prebiotic	capsules	DHA & EPA	108 mg & 1109 mg	1	121*3	56	8.9	67/20 *	WIAT-III/WSCI-III	literacy	+(spelling)	ns	ADHD	
			DHA & EPA	1032 mg & 264 mg	1		54			TEAC	attention and inhibition	+(attention)	ns		
			safflower oil (control)	1467 mg	1		57								
Widenhorn-Müller et al. (2014) [81]	prebiotic	capsules	DHA & EPA	600 mg & 120 mg	1	112	46/49	8.9	74/21	HAWIK-IV	working memory	-	↓	ADHD	
											speed of information processing	-	ns		
										KITAP/TAP	attention	-	ns		
										CBCL AP	attention	NA	ns		
Papalini et al. (2019) [67]	probiotic	powder	Bifidobacterium bifidum W23, Bifidobacterium lactis W51, Bifidobacterium lactis W52, Lactobacillus acidophilus W37, Lactobacillus brevis W63, Lactobacillus casei W56, Lactobacillus salivarius W24, Lactococcus lactis W19, Lactococcus lactis W58	2.5 × 10^9^	2	28	29/29	21.5	0/58	emotional face-matching	emotional reactivity	+	ns	healthy subjects	
										emotional face-word stroop	resolution of emotional conflicts	+	ns		
										color-word stroop	cognitive inhibition	+	ns		
										digit span backward test	working memory	+after stress	↓(after stress)		
Portillo-Reyes et al. (2014) [82]	prebiotic	capsules	DHA & EPA	60 mg & 90 mg	3	91	30/20	9.2	29/21	symbolic search	processing speed	+	↓	healthy children	
										embedded figures test/visual closure	visuoperceptive integration	+	↓		
										semantic fluency/comprehension instruction	language	+semantic fluency (fruit)	ns		
										block design/TMT A	visuomotor coordination	+block design	↓block design		
										matrix reasoning/stroop colour word/TMT B/letter-number sequencing	executive functions	+letter-number seq, matrix reasoning, stroop	↓matrix reasoning, stroop		
										letter cancellation	attention	+	ns		
										rey complex figure/word list	visual & verbal memory	+verbal recall & recognition	ns		
Richardson et al. (2012) [83]	prebiotic	capsules	DHA	200 mg	3	112	179/180	8.6	192/170	BAS-II	reading	+very poor readers only	↓poor and very poor readers only	healthy children	
										DS-FW/DS-BW	working memory	ns	↓ forward recall		
Schmidt et al. (2015) [68]	prebiotic	powder	FOS	5500 mg	1	21	15	23.7	22/23	Attentional dot-probe task	attention	-	↓GOS only, unmasked condition	healthy subjects	
			B-GOS				15			facial expression recognition/emotional word recognition and recall	emotional cognition	-	ns		
			PBO				15								
Steenbergen et al. (2015) [84]	probiotic	powder	Bifidobacterium bifidum W23, Bifidobacterium lactis W51, Bifidobacterium lactis W52, Lactobacillus acidophilus W37, Lactobacillus brevis W63, Lactobacillus casei W56, Lactobacillus salivarius W24, Lactococcus lactis W19, Lactococcus lactis W58	2.5 × 10^9^ CFU	2	28	20/20	19.9	8/32	LEIDS-r	cognitive reactivity to sad mood	+	↓particularly aggressive and ruminative thoughts	healthy subjects	
Vesco et al. (2018) [85]	prebiotic	capsules	DHA & EPA and others	50 mg & 350 mg & 67 mg	2	84	23	11.2	54/41	BRIEF	Executive functions	+	↓	children with depression/mood disorders	
			PUFAs + PEP				22								
			PEP + PBO				26								
			PBO				24								
Voigt et al. (2000) [86]	prebiotic	capsules	DHA	345 mg	3	121	25/24	9.3	42/12 *	TOVA	sustained attention	+	ns	ADHD	
										CCT	visual attention, sequencing, psychomotor speed, cognitive flexibility	ns	ns		
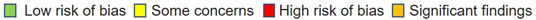															

↓: Improvement vs. placebo; ↑: diminishment vs. placebo; ns: no significant effect; +: improvement of performance vs. baseline; -: not reported or not applicable; dose refers to the amount of active compound. DVT: digit vigilance test, RT: reaction times, DHA: docosahexaenoic acid; EPA: eicosapentaenoic acid; FOS: fructooligosaccharides; B-GOS: Bimuno^®^-galactooligosaccharides; PRO: probiotics; PBO: placebo; RG: reference group; CBCL-AP: Child Behavior Checklist—Attentional Problems; KiTAP: Test of Attentional Performance for Children; TAP: Test of Attentional Performance; CBB: Computerized CogState Brief Battery; RAVLT: Auditory Verbal Learning Test; SCWT: Stroop Color and Word Test; TMT: Trial-Making Test; CANTAB: Cambridge Neuropsychological Test Automated Battery; CDR: Cognitive Drug Research Battery; WIAT: Wechsler Individual Achievement Test; WSCI: Wechsler Scale of Children Intelligence; TEAC: Test of Everyday Attention for Children; HAWIK: Hamburg Wechsler Intelligence Scales for Children, BAS: British Ability Scale; DS-FW: digit-span forward; DS-BW: digit-span backward; LEIDS-r: Leiden Index of Depression Sensitivity—Revised; BRIEF: Behavior Rating Inventory of Executive Functions; TOVA: Test of Variables of Attention; CCT: Children’s Colors Trials test. * As measured at baseline.

## Data Availability

All data supporting the results of this study can be found within the manuscript and supplemental materials.

## Supplementary Materials

The following are available online at https://www.mdpi.com/article/10.3390/nu14030614/s1, Figure S1. Risk of bias evaluation for studies using anxiety and stress outcomes. Figure S2. Risk of bias evaluation for studies using cognitive outcomes.

## Abbreviations

ADHD	Attention-deficit hyperactivity disorder
ASD	Autism spectrum disorder
BAI	Beck Anxiety Inventory
CFU	Colony-forming unit
CIs	Confidence intervals
DASS-42	Depression Anxiety Stress Scale 42
DHA	Docosahexaenoic acid
EPA	Eicosapentaenoic acid
FOS	Fructooligosaccharides
GABA	Gamma-aminobutyric acid
GBA	Gut–brain axis
GOS	Galactooligosaccharides
miRNA	Micro-RNA
mRNA	Messenger RNA
PGN	Peptidoglycan
PSWQ	Penn State Worry Questionnaire
PUFAs	Omega-3-polyunsaturated fatty acids
RoB-2	Risk of bias tool for randomization trials
SCFAs	Short-chain fatty acids
STAI	State-Trait Anxiety Inventory
VAS	Visual Analogue Scale
