# Supplementary material for: A Systematic Review of Psychobiotic Interventions in Children and Adolescents to Enhance Cognitive Functioning and Emotional Behavior
Source: Nutrients. 2022 Jan 30;14(3):614. doi: 10.3390/nu14030614 (PMC8840038; doi:10.3390/nu14030614)
Supplement: Supplementary file 1 [file nutrients-14-00614-s001.zip › Supplemental material_Basso et al.pdf]

Supplemental material

A Systematic Review of Psychobiotic Interventions in Children and Adolescents to Enhance Cognitive Functioning and Emotional Behavior

Melissa Basso[1], Nicola Johnstone[1],*, Paul Knytl[1], Arjen Nauta[2], Andre Groeneveld[2] and Kathrin Cohen Kadosh[1],*

[1]Department of Psychological Sciences, School of Psychology, Faculty of Health and Medical Scienc-es, University of Surrey, Guildford GU2 7XH, UK; m.basso@surrey.ac.uk (M.B.); p.knytl@surrey.ac.uk (P.K.)

[2]FrieslandCampina, 3818 LE Amersfoort, The Netherlands; arjen.nauta@frieslandcampina.com (A.N.); andre.groeneveld@frieslandcampina.com (A.G.)

*Correspondence: nicola.johnstone@surrey.ac.uk (N.J.); k.cohenkadosh@surrey.ac.uk (K.C.K.); Tel.: +44 -(0)-1483-68-3968 (K.C.K.)

Supplemental Methods

Protocol

PRISMA checklist

Section and Topic	Item #	Checklist item	Location where item is reported
**TITLE**			
Title	1	Identify the report as a systematic review.	1
**ABSTRACT**			
Abstract	2	See the PRISMA 2020 for Abstracts checklist.	1
**INTRODUCTION**			
Rationale	3	Describe the rationale for the review in the context of existing knowledge.	1-4
Objectives	4	Provide an explicit statement of the objective(s) or question(s) the review addresses.	4
**METHODS**			
Eligibility criteria	5	Specify the inclusion and exclusion criteria for the review and how studies were grouped for the syntheses.	4
Information sources	6	Specify all databases, registers, websites, organisations, reference lists and other sources searched or consulted to identify studies. Specify the date when each source was last searched or consulted.	4
Search strategy	7	Present the full search strategies for all databases, registers and websites, including any filters and limits used.	Supp. methods, Search strategy.
Selection process	8	Specify the methods used to decide whether a study met the inclusion criteria of the review, including how many reviewers screened each record and each report retrieved, whether they worked independently, and if applicable, details of automation tools used in the process.	4
Data collection process	9	Specify the methods used to collect data from reports, including how many reviewers collected data from each report, whether they worked independently, any processes for obtaining or confirming data from study investigators, and if applicable, details of automation tools used in the process.	4
Data items	10a	List and define all outcomes for which data were sought. Specify whether all results that were compatible with each outcome domain in each study were sought (e.g. for all measures, time points, analyses), and if not, the methods used to decide which results to collect.	4
	10b	List and define all other variables for which data were sought (e.g. participant and intervention characteristics, funding sources). Describe any assumptions made about any missing or unclear information.	4
Study risk of bias assessment	11	Specify the methods used to assess risk of bias in the included studies, including details of the tool(s) used, how many reviewers assessed each study and whether they worked independently, and if applicable, details of automation tools used in the process.	4-5
Effect measures	12	Specify for each outcome the effect measure(s) (e.g. risk ratio, mean difference) used in the synthesis or presentation of results.	-
Synthesis methods	13a	Describe the processes used to decide which studies were eligible for each synthesis (e.g. tabulating the study intervention characteristics and comparing against the planned groups for each synthesis (item #5)).	4
	13b	Describe any methods required to prepare the data for presentation or synthesis, such as handling of missing summary statistics, or data conversions.	-
	13c	Describe any methods used to tabulate or visually display results of individual studies and syntheses.	-
	13d	Describe any methods used to synthesize results and provide a rationale for	4

Section and Topic	Item #	Checklist item	Location where item is reported
		the choice(s). If meta-analysis was performed, describe the model(s), method(s) to identify the presence and extent of statistical heterogeneity, and software package(s) used.	
	13e	Describe any methods used to explore possible causes of heterogeneity among study results (e.g. subgroup analysis, meta-regression).	-
	13f	Describe any sensitivity analyses conducted to assess robustness of the synthesized results.	-
Reporting bias assessment	14	Describe any methods used to assess risk of bias due to missing results in a synthesis (arising from reporting biases).	-
Certainty assessment	15	Describe any methods used to assess certainty (or confidence) in the body of evidence for an outcome.	-
**RESULTS**			
Study selection	16a	Describe the results of the search and selection process, from the number of records identified in the search to the number of studies included in the review, ideally using a flow diagram.	5, 9
	16b	Cite studies that might appear to meet the inclusion criteria, but which were excluded, and explain why they were excluded.	/
Study characteristics	17	Cite each included study and present its characteristics.	5-7, 9-11
Risk of bias in studies	18	Present assessments of risk of bias for each included study.	8, 12, Supp. Results.
Results of individual studies	19	For all outcomes, present, for each study: (a) summary statistics for each group (where appropriate) and (b) an effect estimate and its precision (e.g. confidence/credible interval), ideally using structured tables or plots.	10, 13
Results of syntheses	20a	For each synthesis, briefly summarise the characteristics and risk of bias among contributing studies.	9-10, 12-13
	20b	Present results of all statistical syntheses conducted. If meta-analysis was done, present for each the summary estimate and its precision (e.g. confidence/credible interval) and measures of statistical heterogeneity. If comparing groups, describe the direction of the effect.	8, 12-13
	20c	Present results of all investigations of possible causes of heterogeneity among study results.	13
	20d	Present results of all sensitivity analyses conducted to assess the robustness of the synthesized results.	-
Reporting biases	21	Present assessments of risk of bias due to missing results (arising from reporting biases) for each synthesis assessed.	-
Certainty of evidence	22	Present assessments of certainty (or confidence) in the body of evidence for each outcome assessed.	-
**DISCUSSION**			
Discussion	23a	Provide a general interpretation of the results in the context of other evidence.	14-16
	23b	Discuss any limitations of the evidence included in the review.	14-16
	23c	Discuss any limitations of the review processes used.	14-16
	23d	Discuss implications of the results for practice, policy, and future research.	15-16
**OTHER INFORMATION**			
Registration and protocol	24a	Provide registration information for the review, including register name and registration number, or state that the review was not registered.	4
	24b	Indicate where the review protocol can be accessed, or state that a protocol was not prepared.	4
	24c	Describe and explain any amendments to information provided at registration or in the protocol.	-

Section and Topic	Item #	Checklist item	Location where item is reported
Support	25	Describe sources of financial or non-financial support for the review, and the role of the funders or sponsors in the review.	17
Competing interests	26	Declare any competing interests of review authors.	17
Availability of data, code and other materials	27	Report which of the following are publicly available and where they can be found: template data collection forms; data extracted from included studies; data used for all analyses; analytic code; any other materials used in the review.	4, 5, 8, 9, 12,17

Search strategy

Search String Terms and Databases Searched:

1 PUBMED

#1 ((((((((humans [MeSH Terms]) OR patients [MeSH Terms]) OR research subjects [MeSH Terms]) OR human experimentation [MeSH Terms]) OR human * [Abstract/title]) OR client * [Abstract/title]) OR individual * [Abstract/title]) OR subject * [Abstract/title]) OR participant * [Abstract/title].

#2 ((((((((((((minors [MeSH Terms]) OR adolescent [MeSH Terms]) OR child, development [MeSH Terms]) OR paediatric [MeSH Terms]) OR puberty [MeSH Terms]) OR young adult [MeSH Terms]) OR teen * [Abstract/Title]) OR young men [Abstract/title]) OR young women [Abstract/title]) OR adolescen * [Abstract/title]) OR youth [Abstract/title]) OR undergraduate student * [Abstract/title]) OR college student * [Abstract/title].

#3 ((((((((((probiotics [MeSH Terms]) OR prebiotics [MeSH Terms]) OR dietary carbohydrates [MeSH Terms]) OR dietary fiber [MeSH Terms]) OR psychobiotic [Abstract/title]) OR probio*[Abstract/title]) OR prebio * [Abstract/title]) OR pro-bio * [Abstract/title]) OR pre-bio * [Abstract/title]) OR lactobacill * [Abstract/title]) OR bifidobacteri * [Abstract/title].

#4 ((((((((((((((((((((((((((anxiety [MeSH Terms]) OR anxiety disorders [MeSH Terms]) OR test anxiety scale [MeSH Terms]) OR manifest anxiety scale [MeSH Terms]) OR patient health questionnaire [MeSH Terms]) OR (survey [MeSH Terms]) AND questionnaire/psychology [MeSH Terms])) OR psychology, child [MeSH Terms]) OR psychology, adolescent [MeSH Terms]) OR child behavior disorders [MeSH Terms]) OR panic [MeSH Terms]) OR affect [MeSH Terms]) OR affective symptoms [MeSH Terms]) OR performance anxiety [MeSH Terms]) OR stress, psychological [MeSH Terms]) OR psychological stress [MeSH Terms]) OR emotions [MeSH Terms]) OR emotion regulation [MeSH Terms]) OR anxi * [Abstract/title]) OR stress [Abstract/title]) OR PSS [Abstract/title]) OR HADS [Abstract/title]) OR SAS [Abstract/title]) OR DASS [Abstract/title]) OR HAD-S [Abstract/title]) OR PHQ [Abstract/title]) OR BAI [Abstract/title]) OR STAI [Abstract/title]) OR emotional decision * [Abstract/title].

#5 ((((((((((((((((((((((cognition [MeSH Terms]) OR attention [MeSH Terms]) OR memory [MeSH Terms]) OR executive functions [MeSH Terms]) OR learning [MeSH Terms]) OR neuropsychological test [MeSH Terms]) OR cogni * [Abstract/title]) OR processing [Abstract/title]) OR Cambridge Neuropsychological Test Automated Battery [Abstract/title]) OR CANTAB [Abstract/title]) OR Flanker Task [Abstract/title]) OR attention network [Abstract/title]) OR ANT [Abstract/title]) OR stroop [Abstract/title]) OR N-back [Abstract/title]) OR go-no-go [Abstract/title]) OR stop signal [Abstract/title]) OR delay of gratification [Abstract/title]) OR digit span [Abstract/title]) OR automated working memory assessment battery [Abstract/title]) OR AWMA [Abstract/title]) OR AX continuous performance task [Abstract/title]) OR AX-CPT [Abstract/title]) OR dot-probe [Abstract/title].

#1 and #2 and #3 and #4 = 82 studies.

#1 and #2 and #3 and #5 = 98 studies.

2 COCHRANE

#1 [mh humans] OR [mh patients] or [mh "research subjects"] OR [mh "human experimentation"] OR participant: ti, ab, kw OR client:ti,ab, kw OR individual: ti, ab, kw or subject: ti, ab, kw.
#2 [mh minors] OR [mh adolescent] OR [mh adolescence] OR [mh child] or [mh puberty] or [mh "adolescent, development"] or [mh "child, development"] OR [mh paediatric] or "young men": ti, ab, kw or "young women": ti, ab, kw or teen *: ti, ab, kw or adolescen *: ti, ab, kw or "undergraduate student": ti, ab, kw or "college student": ti, ab, kw.
#3 [mh probiotics] or [mh prebiotics] or psychobiotic * or probio * or prebio * or [mh "dietary carbohydrates"] or [mh "dietary fiber"] or lactobacill *: ti, ab, kw or bifidobacter *: ti, ab, kw.
#4 [mh anxiety] or [mh "anxiety disorders"] or [mh affect] or [mh panic] or [mh "affective symptoms"] or [mh "patient health questionnaire"] or [mh "survey and questionnaires"/PX] or [mh "psychology, child"] or [mh "psychology, adolescent"] or [mh "test anxiety scale"] or [mh "manifest anxiety scale"] or [mh "child behavior disorders"] or [mh "performance anxiety"] or [mh "stress, psychological"] or [mh "emotion regulation"] or [mh emotions] or [mh "psychological distress"] or anxi *: ti, ab, kw or sas: ti, ab, kw or dass: ti, ab, kw or hads: ti, ab, kw or had-s: ti, ab, kw or stai: ti, ab, kw or bai: ti, ab, kw or phq: ti, ab, kw or pss: ti, ab, kw.
#5 [[mh cognition] or [mh attention] or [mh memory] or "executive function":ti, ab or "cogni *": ti, ab or processing: ti, ab or "Cambridge Neuropsychological Test Automated Battery": ti, ab or CANTAB: ti, ab or "Flanker test": ti, ab or "attention network": ti, ab or ANT: ti, ab or stroop: ti, ab or "Automated Working Memory Assessment battery": ti, ab or AWMA: ti, ab or "AX continuous performance": ti, ab or "AX CPT": ti, ab or "dot probe": ti, ab.

#1 and #2 and #3 and #4 = 25 reviews, 654 trials.

#1 and #2 and #3 and #5 = 8 reviews, 104 trials.

3 MEDLINE

#1 (MM "Humans") OR (MH "Patients+") OR (MH "Research Subjects+") OR (MH "Human Experimentation+") OR "human *" OR "subject *" OR "individual * OR "client *" OR participant *.
#2 (MM "Minors") OR (MM "Adolescent") OR (MM "Adolescent Development") OR (MH "Child+") OR (MH "Child Development+") OR "adolescen *" OR (MH "Pediatrics") OR (MM "Puberty") OR (MM "Young Adult") OR "teen *" OR "youth" OR "young men" OR "young women" or "undergraduate student" or "college student".
#3 (MM "Probiotics") OR (MM "Prebiotics") OR (MM "Dietary Carbohydrates") OR (MM "Dietary Fiber") OR "psychobiotic *" OR "probio * OR "prebio *" OR "pro-bio *" OR "pre-bio *" OR "lactobacill *" OR "bifidobacteri *".
#4 (MH "Anxiety+") OR (MH "Anxiety Disorders+") OR (MM "Test Anxiety Scale") OR (MM "Manifest Anxiety Scale") OR (MM "Patient Health Questionnaire") OR (MM "Surveys and Questionnaires"/PX) OR (MM "Psychology, Child") OR (MM "Psychology, Adolescent") OR (MM "Child Behavior Disorders") OR (MM "Panic") OR (MH "Affect+") OR (MH "Affective Symptoms+") OR (MM "Performance Anxiety") OR (MH "Stress, Psychological+") OR (MH "Emotions+") OR (MH "Psychological Distress") OR (MM "Emotional Regulation") OR "SAS" OR "DASS" OR "HADS" OR "HAD-S" OR "BAI" OR "STAI" OR "PHQ" OR "PSS" OR anxi * OR "Emotional decision *" OR stress.

#5 (MM “Cognition”) OR (MM “Cognition disorders”) OR (MM “attention”) OR (MM “attentional bias”) OR (MM “memory”) OR (MM “memory disorders”) OR (MM “executive function”) OR (MM “neuropsychological tests”) OR “cogni *” OR “processing or Cambridge Neuropsychological Test Automated Battery” or “CANTAB” or “Flanker Task” or “Attention Network” or “ANT” or “Stroop” or “N-back” or “Go-no-go” or “Stop signal” or “Delay of gratification” or “Digit span” or “Automated Working Memory Assessment battery” or “AWMA” or “AX continuous performance task” or AX-CPT or “dot-probe”.

#1 and #2 and #3 and #4 = 111 studies.

#1 and #2 and #3 and #5 = 57 studies.

4 SCOPUS

#1 (TITLE-ABS-KEY (human OR patient OR “research subject” OR “human trial” OR subject OR client OR individual)) AND (TITLE-ABS-KEY (minor OR adolescen! OR child! OR “young adult” OR teen! OR “young m?n” OR “young wom?n” OR youth OR “undergraduate student *” OR “college student*”)) AND (TITLE-ABS-KEY (probiotic OR prebiotic OR “dietary fiber” OR psychobiotic OR lactobacill! OR bifidobacteri!)) AND (TITLE-ABS-KEY (anxi! OR panic OR psychologic! OR affect OR “emotion regulation” OR “emotional disorder” OR “Patient health questionnaire” OR sas OR dass OR had?s OR bai OR stai OR phq OR stress OR distress)).

#2 (TITLE-ABS-KEY (human OR patient OR “research subject” OR “human trial” OR subject OR client OR individual)) AND (TITLE-ABS-KEY (minor OR adolescen! OR child! OR “young adult” OR teen! OR “young m?n” OR “young wom?n” OR youth OR “undergraduate student*” OR “college student *”)) AND (TITLE-ABS-KEY (probiotic OR prebiotic OR “dietary fiber” OR psychobiotic OR lactobacill! OR bifidobacteri!)) AND ((TITLE-ABS-KEY (cogni! OR attention! OR “executive function” OR memory OR “neuropsychological test” OR processing OR “Cambridge Neuropsychological Test Automated Battery” OR cantab OR “flanker task” OR “attention! Network” OR ant OR “N-back” OR “go-no-go” OR “stop signal”) OR TITLE-ABS-KEY (“delay of gratification” OR “digit span” OR “Automated Working Memory Assessment battery” OR awma OR “AX continuous performance task” OR ax-cpt))).

#1 = 982 studies.

#2 = 426 studies.

5 EMBASE

#1 “human”/exp OR “patient”/exp OR “research subject”/exp OR “human experiment”/exp OR human *: ti, ab OR client *: ti, ab OR individual *: ti, ab OR subject *: ti, ab OR participant *: ti, ab.

#2 “minor”/exp OR “adolescent”/exp OR “child”/exp OR “adolescence”/exp OR “young adult”/exp OR “young m#n”: ti, ab OR “young wom#n”: ti, ab OR teen *: ti, ab OR adolescen *: ti, ab OR youth: ti, ab or “undergraduate student *”: ti, ab or “college student *”: ti, ab.

#3 “probiotic agent”/exp OR “prebiotic agent”/exp OR “dietary fiber”/exp OR “psychobiotic agent”/exp OR “lactobacillus”/exp OR “7ambridge7erium”/exp OR psychobiotic *: ti, ab OR pro?bio *: ti, ab OR pre?bio *: ti, ab OR lactobacill *: ti, ab OR bifidofacteri *: ti, ab.

#4 "anxiety"/exp OR "anxiety disorder"/exp OR "anxiety psychology"/exp OR "anxiety assessment"/exp OR "child psychology"/exp OR "affect"/exp OR "emotion regulation"/exp OR "emotional disorder"/exp OR "emotional stress"/exp OR "patient health questionnaire"/exp OR "behavior/psychological aspect" OR sas: ti, ab OR dass: ti, ab OR had?s: ti, ab OR bai: ti, ab OR stai: ti, ab OR phq: ti, ab OR pss: ti, ab OR anxi *: ti, ab OR distress :ti, ab OR stress: ti, ab OR "emotional decision *": ti, ab.

#5 "cognition"/exp OR "cognitive defect"/exp OR "memory"/exp OR "neuropsychological test"/exp OR "attention"/exp OR "attentional bias"/exp OR cogni *: ti, ab OR processing: ti, ab OR "8ambridge neuropsychological test automated battery": ti, ab OR cantab: ti, ab OR "flanker task": ti, ab OR "attention network": ti, ab OR ant: ti, ab OR stroop: ti, ab OR "n-back": ti, ab OR "go-no-go": ti, ab OR "stop signal": ti, ab OR "delay of gratification": ti, ab OR "digit span": ti, ab OR "automated working memory assessment battery": ti, ab OR awma: ti, ab OR "ax continuous performance task": ti, ab OR "ax-cpt": ti, ab OR "dot probe": ti, ab.

#1 and #2 and #3 and #4 = 377 studies.

#1 and #2 and #3 and #5 = 560 studies.

6 PSYCHINFO:

#1 DE "Experimental Subjects" OR AB participant * OR AB subject * OR AB individual* OR AB human *.

#2 DE "Adolescent Psychopathology" OR DE "Adolescent Psychology" OR DE "Adolescent Psychiatry" OR DE "Adolescent Development" OR DE "Adolescent Behavior" OR DE "Adolescent Characteristics" OR DE "Adolescent Health" OR DE "Early Adolescence" OR AB child * OR AB adolescen * OR AB teen* OR AB youth OR AB "young adult *" OR AB "young women" OR AB "young men" or AB"undergraduate student *" or AB"college student *".

#3 DE "Dietary Supplements" OR AB probio * OR AB prebio * OR AB lactobacill * OR AB bifidobacteri * OR AB psychobiotic * OR AB "dietary fiber *".

#4 DE "Anxiety" OR DE "Anxiety Sensitivity" OR DE "Computer Anxiety" OR DE "Health Anxiety" OR DE "Mathematics Anxiety" OR DE "Performance Anxiety" OR DE "Social Anxiety" OR DE "Speech Anxiety" OR DE "Test Anxiety" OR DE "Anxiety Disorders" OR DE "Castration Anxiety" OR DE "Death Anxiety" OR DE "Generalized Anxiety Disorder" OR DE "Obsessive Compulsive Disorder" OR DE "Panic Attack" OR DE "Panic Disorder" OR DE "Phobias" OR DE "Separation Anxiety Disorder" OR DE "Trichotillomania" OR DE "Taylor Manifest Anxiety Scale" OR DE "State Trait Anxiety Inventory" OR DE "Children Manifest Anxiety Scale" OR DE "Child Behavior Checklist" OR DE "Mental Health and Illness Assessment" OR DE "Health Psychology Assessment" OR DE "Health Attitude Measures" OR DE "Health Behavior Measures" OR DE "Psychodiagnostic Measures" OR DE "Psychodiagnostic Interview" OR DE "Diagnostic Interview Schedule" OR DE "Structured Clinical Interview" OR DE "Attentional Bias" OR DE "Behavioral Assessment" OR DE "Distress" OR AB anxi* or AB "emotion regulation" OR AB SAS OR AB DASS OR AB DA?S OR AB BAI OR AB STAI OR AB PHQ OR AB PSS.

#5 DE "Cognition" OR DE "cognitive ability" OR DE "cognitive assessment" or DE "attention" OR DE "attentional bias" OR DE "memory" OR DE "memory disorders" OR DE "executive function" OR DE "neuropsychology" or AB cogni*OR AB "processing or Cambridge Neuropsychological Test Automated Battery" or AB CANTAB or AB "Flanker Task" or AB "Attention Network" or AB ANT or AB Stroop or

AB “N-back” or AB “Go-no-go” or AB “Stop signal” or AB “Delay of gratification” or AB “Digit span” or AB “Automated Working Memory Assessment battery” or AB AWMA or AB “AX continuous performance task” or AB “AX-CPT” or AB “dot-probe.”

#1 and #2 and #3 and #4 = 46 studies.

#1 and #2 and #3 and #5 = 31 studies.

7 WEB OF SCIENCE

#1 TOPIC (human * OR patient * OR subject * OR client * OR individual *).

#2 TOPIC (adolescen * OR child * OR “young adult *”OR teen * OR “young m * n” OR “young wom * n” OR youth or “undergraduate student *” or “college student *”).

#3 TOPIC (prebiotic * OR probiotic * OR psychobiotic *OR lactobacill * OR bifidobacter * OR “dietary fiber *”).

#4 TOPIC (anxi * OR affect * OR “emotion * regulation” OR “emotional disorder *” OR “patient health questionnaire” OR sas OR dass OR had?s OR stai OR bai OR phq OR pss OR panic OR “psychological distress” OR stress).

#5 TOPIC (cogni * OR memory OR attention * OR “executive function *” OR “neuropsychological test *” OR “processing or Cambridge Neuropsychological Test Automated Battery” or CANTAB or “Flanker Task” or “Attention Network” or ANT or Stroop or “N-back” or “Go-no-go” or “Stop signal” or “Delay of gratification” or “Digit span” or “Automated Working Memory Assessment battery” or AWMA or “AX continuous performance task” or AX-CPT or “dot probe”)

#1 and #2 and #3 and #4 = 111 studies.

#1 and #2 and #3 and #5 = 85 studies.

Supplemental results

Quality of included studies

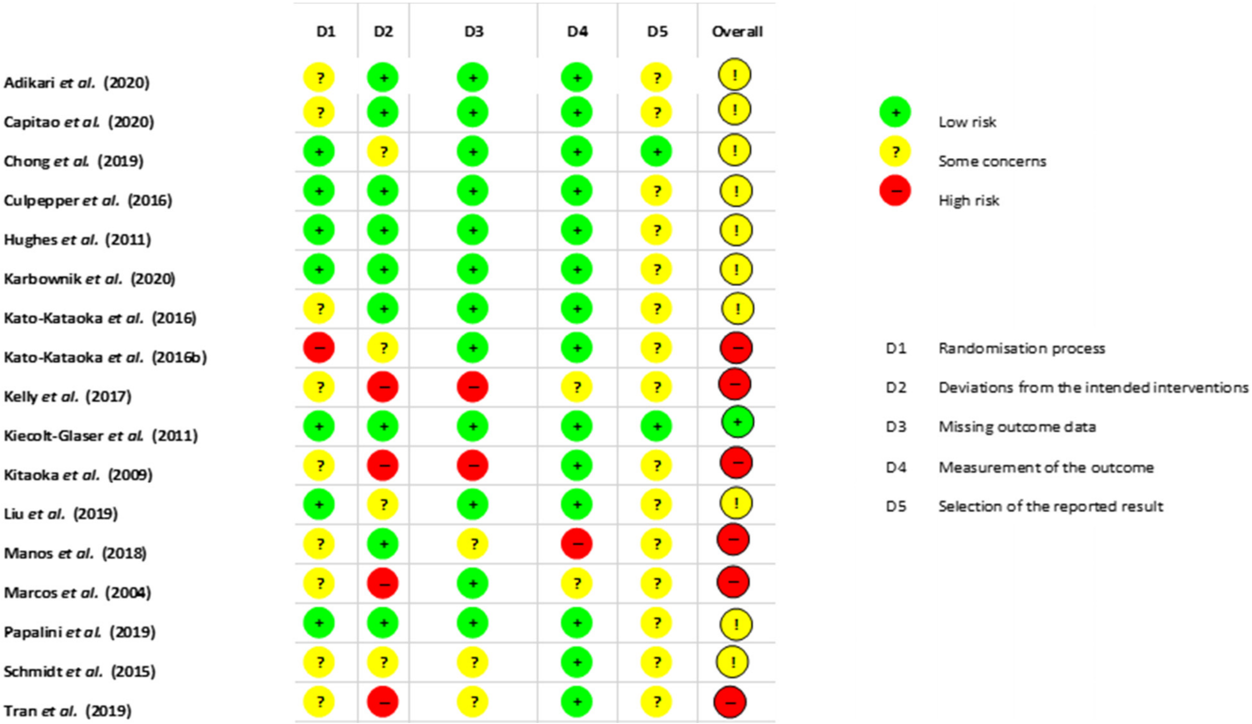

Figure S1. Risk of bias evaluation for studies using anxiety and stress outcomes.

	D1	D2	D3	D4	D5	Overall
Adikari *et al.* (2020)	!	+	+	+	!	!
Bos *et al.* (2015)	!	+	+	+	!	!
Cornu *et al.* (2018)	+	+	+	+	+	+
Chong *et al.* (2019)	+	!	+	+	+	!
Karr Justin *et al.* (2012)	+	!	+	+	!	!
Kelly *et al.* (2017)	!	-	-	!	!	-
Kennedy *et al.* (2009)	+	!	+	+	!	!
Liu *et al.* (2019)	+	+	+	+	!	!
Milte *et al.* (2012)	+	+	+	+	!	+
Milte *et al.* (2013)	+	+	+	+	!	+
Muller *et al.* (2014)	+	+	+	+	!	+
Papalini *et al.* (2018)	+	+	+	+	!	!
Portillo-Reyes *et al.* (2014)	!	!	+	!	!	!
Richardson *et al.* (2012)	+	+	+	+	+	+
Schmidt *et al.* (2015)	!	+	+	+	!	!
Steenbergen *et al.* (2015)	+	+	+	+	!	!
Vesco *et al.* (2018)	!	+	!	+	-	-
Voigt *et al.* (2000)	+	+	+	+	!	!
Capitao *et al.* (2020)	!	+	+	+	!	!

+ Low risk
! Some concerns
- High risk

D1 Randomisation process
D2 Deviations from the intended interventions
D3 Missing outcome data
D4 Measurement of the outcome
D5 Selection of the reported result

Figure S2. Risk of bias evaluation for studies using cognitive outcomes.
